# Amyloid precursor-like protein 2 interacts with claudin-7 and affects ovarian cancer cell survival

**DOI:** 10.2144/fsoa-2019-0123

**Published:** 2020-03-16

**Authors:** Neetu Dahiya

**Affiliations:** 1 Laboratory of Molecular Biology & Immunology, NIH, Baltimore, MD 21224, USA; 2 Center for Biologics Evaluation & Research, US Food & Drug Administration, Silver Spring, MD 20993, USA

**Keywords:** APLP2, CLDN7, ovarian cancer, yeast two-hybrid system

## Abstract

**Aim:**

In our previous report, we identified roles of CLDN7 in regulation of cell signaling. The goal of this study was to identify proteins interacting with CLDN7 in ovarian cancer.

**Methods:**

The yeast two-hybrid system was used to identify proteins directly interacting with CLDN7 and cell survival was tested using colony formation assay.

**Results:**

Amyloid precursor-like protein 2 (APLP2) was found directly associated with CLDN7 in ovarian cancer cell line OVCA420. In addition, APLP2 showed increased expression in ovarian cancer cell lines and tumor tissue samples compared with non-neoplastic ovarian tissues. Knockdown of CLDN7 led to increased expression of APLP2 at both the mRNA and protein levels. Knockdown of APLP2 was associated with decreased cell survival in ovarian cancer cells.

**Conclusion:**

We show a direct interaction of CLDN7 with APLP2. These findings suggest novel regulatory role for APLP2 in ovarian cancer, a role that appears to be mediated by CLDN7.

## Lay abstract

We conducted this study to find other proteins interacting with CLDN7. CLDN7 is a tight junction protein and increased expression of CLDN7 has been reported in several cancers, including ovarian cancer. Using a yeast two-hybrid approach, we identified amyloid precursor-like protein 2 (APLP2) directly interacting with CLDN7. Like CLDN7, increased levels of APLP2 proteins were found in both ovarian cancer cell lines and ovarian cancer tissue samples. We also identified functional role of APLP2 in ovarian cancer where lowering APLP2 protein levels led to decreased cell survival in ovarian cancer cell line, OVCA420.

Tight junctions (TJ) are a type of cell–cell adhesion, which is involved in the formation of ion and size-selective barriers between epithelial and endothelial cells. Different proteins associated with these junctions are involved in different molecular functions such as regulation of cellular polarity, cellular proliferation, transcription and barrier functions in the cells. Different components of TJ can be categorized as: integral TJ proteins, which forms a regulated permeability barrier, PDZ (post-synaptic density protein [PSD95], *Drosophila* disc large tumor suppressor [Dlg1], and zonula occludens-1 protein [zo-1]) domains, which are involved in interaction of integral TJ proteins with the actin cytoskeleton and other cell signaling proteins, cytosolic or nuclear proteins associated indirectly or directly with TJ proteins and are involved in the regulation of tight junction function such as paracellular transport, cell polarity, cell proliferation etc. [1]. The major proteins of tight junctions are occludins, claudins, junctional adhesion molecule 1, zona occludens 1, multiple PDZ domain protein 1 and cingulin. Claudins are important structural components of tight junctions which are required for ion selective pore formation. Claudins are 20–24 kDa multidomain proteins comprising of four transmembrane domains, two extra cellular loops and cytosolic N- and C-termini. Claudin proteins have been implicated in regulating tight junction functions. Tight junctions are compromised in various disease conditions including a variety of cancers, such as breast cancer [2], ovarian cancer [3] and lung cancer [4]. A total of 24 claudins have been characterized in humans, with the function being determined by the type of claudin protein present in the tight junction. For example, higher expression of claudin-2 in MDCK II is responsible for weaker tight junction compared with MDCK I cell line expressing mainly claudin-1 and claudin-4 [5].

Our laboratory is focused on understanding the role of claudin proteins in ovarian cancer [3]. In our previous study, we demonstrated that CLDN7 is increased in ovarian cancer cell lines and tissues at both mRNA and protein levels [6]. SiRNA-mediated downregulation of CLDN7 led to changes in the expression of several genes at mRNA and protein levels, indicating that, similar to some tight junction-associated proteins, such as ZO-1, ZO-2, ZONAB [7] and CLDN7, may regulate gene expression. We decided to investigate any interacting partners of CLDN7 which might be involved in CLDN7-mediated signaling. To identify the CLDN7 interacting partners, we used split-ubiquitin membrane-based yeast two-hybrid system. It is based on split ubiquitin assay which can detect interaction of membrane proteins with other integral membrane proteins, membrane-associated proteins or cytosolic proteins [8]. The aim of the current study was to find interacting partner(s) of CLDN7 in ovarian cancer. This may shed light on the mechanisms and roles of CLDN7 in the development and progression of ovarian cancer.

## Materials & methods

### Yeast strain, plasmids, cDNA library, tissues & cell lines

Yeast two-hybrid screening was performed in the yeast reporter strain, NMY51 (Dualsystems Biotech, Schlieren, Switzerland). CLDN7 was cloned in plasmid pBT3-STE (X-Cub LexA VP16) for serving as the bait vector. The prey vectors were generated in plasmid pPR3-N (NubG-X) from the human adult kidney cDNA library. We used three different control vectors: pCCW-Alg5-control bait vector, pDL2-Alg5-negative control prey vector and pAI-Alg5-positive control prey vector for testing our bait vector (pBT3-STE-CLDN7) and optimizing the conditions for cDNA library screen. All human tissues were collected from MedStar Research Institute (MD, USA) under approved IRB protocol (IRB#2003-103). All the ovarian cancer cell lines used in this study have been derived from serous epithelial ovarian cancers and published in literature [9–11]

### Construction of the bait vector

The full length *CLDN7* cDNA was cloned in pBT3-STE vector. *CLDN7* cDNA was amplified from OVCA420 cell line cDNA using (ATTAACAA*GGCCATTACGGCC*ACCATGGCCAATTCGGGCC- forward) and (AACTGATT*GGCCGAGGCGGCC*CACACATACTCCTTGGAAGAGTT-reverse) primers and inserted at *Sfi1*sites in pBT3-STE vector. The stop codon was removed from pBT3-STE to make a fusion protein, in other words, CLDN7-Cub LexA-VP16.

### Yeast two-hybrid screening

The yeast reporter strain NMY51 was transformed with the bait vector using a standard Polyethylene glycol (PEG)/lithium acetate-based protocol and expression of the bait was confirmed by immunoblotting using LexA antibody (Dualsystems). Lack of self-activation of bait was tested by cotransformation with empty library vector, in other words, pPR3-N in NMY51 and examining growth on selective media. Additional controls were performed by cotransforming a control bait vector (pCCW-Alg5-control) with the pDL2-Alg5-negative control prey vector and pAI-Alg5-positive control prey vector. To avoid false positives in our screen, we optimized the screening stringency by incorporating 3-aminotriazol (3-AT, a competitive inhibitor of *HIS3* gene product) in the selective plates.

Human cDNA kidney library was transformed into NMY51 cells carrying the bait vector, in other words, pBT3-STE-CLDN7-LexA-VP16 and selected on SD Leu-Trp-His-Ademedium containing 75 mM 3-AT. The transformants were screened for lacZ activation by b-galactosidase using filter lift-off assay. Plasmids were isolated using lyticase followed by Qiagen maxiprep method and transformed in *E. coli* using standard protocol. Plasmids were run on gel and tested for presence of insert using *Sfi1* digestion. Protein preparation from yeast was performed using protein isolation kit from Dualsystems Biotech.

### Immunofluorescence

OVCA420 cell expressing CLDN7 and amyloid precursor-like protein 2 (APLP2) were grown on four chamber slides for 72 h. Cells were washed with phosphate buffered saline (PBS) containing 1 mM CaCl_2_ and 1 mM MgCl_2_, followed by fixing in cold methanol at -20°C for 10 min. Then cells were washed with PBS and blocked and permeabilized with 5% bovine serum albumin and 0.2% Triton X-100 at room temperature for 30 min. The cells were incubated with antimouse CLDN7 antibody (1:100) or antirabbit APLP2 antibody (1:100) for 1 h at 37°C. After three washes in wash buffer (PBS with 0.05% Triton X-100) cells were incubated with secondary fluorescent antibodies, Alexa Fluor 488 mouse and Alexa Fluor 555 rabbit for CLDN7 and APLP2, respectively, for 1 h at 37°C in dark. Cells were washed three-times with wash buffer, rinsed twice with PBS and mounted in DAPI containing Pro Long Gold antifade reagent (Thermo Fisher, CA, USA).

### Immunoprecipitation

For immunoprecipitation (IP) studies, OVCA420 cells were grown for 72 h and washed with ice-cold PBS. Lysates were prepared using Pierce lysis buffer (25 mM Tris-HCl pH 7.4, 150 mM NaCl, 1 mM EDTA, 1% NP-40 & 5% glycerol). After incubating for 1 h on ice, lysates were centrifuged at 14,000 rpm for 10 min at 4°C. After overnight incubation of lysates, with CLDN7 or APLP2 or GAPDH antibody, 50 μl Protein A and Protein G beads were added (1:1) followed by a 2 h incubation at 4°C under rotation. The beads were washed three-times with lysis buffer followed by boiling in SDS-PAGE sample buffer for 5 min.

### siRNA-mediated knockdown of CLDN7 & APLP2

siRNAs for *CLDN7* targeting were purchased from Ambion (Ambion/Applied Biosystem, CA, USA). For *APLP2* knockdown, APLP2 specific ON-TARGET Plus siRNAs were purchased from Dharmacon (IL, USA). OVCA420 cells were transfected with CLDN7 and APLP2 siRNAs (100 ng/well in a six-well dish) using Lipofectamine-2000 (Invitrogen, CA, USA) as per manufacturer’s instructions. Nonspecific siRNA from Dharmacon was used as a negative control. After 48–72 h, cells were harvested for immunoblotting, RNA preparation or used for other assays.

### Real-time PCR

Total RNA was prepared using Trizol (Invitrogen) according to the manufacturer’s instructions. RNA was quantified using the RNA 6000 Nano Kit (Agilent Technologies, CA, USA). Reverse transcription polymerase chain reaction (RT-PCR) was performed as previously described [12]. A total of 1 μg of RNA was obtained from the various tissues and cell lines using Trizol (Invitrogen), which was used to generate cDNA using Taqman Reverse Transcription Reagents (PE Applied Biosystems, CA, USA). The SYBR Green I assay and the GeneAmp 5700 Sequence Detection System (PE Applied Biosystems) were used for detecting real-time PCR products. The PCR cycling conditions were performed for all samples as follows: 50°C, 2 min for AmpErase UNG incubation; 95°C, 10 min for AmpliTaq Gold activation; and, 40 cycles for the melting (95°C, 15 s) and annealing/extension (60°C for 1 min) steps. PCR reactions for each template were done in duplicate in 96-well plates. The comparative CT method (PE Applied Biosystems) was used to determine gene expression in each sample relative to the value observed in the nonmalignant HOSE-B, using GAPDH as normalization control. Quantitative polymerase chain reaction (Q-PCR) was performed using following primers: APLP2 (forward 5′-CTGATTCAGCACTTCCAAGC-3′; reverse 5′-GGTAGTTCTCCAGAGCCATC-3′), CLDN7 (forward, 5′-AGAGCACGGGGATGATGAG-3′; reverse, 5′-CACCCATGGCTATACGGGC-3′) and GAPDH (forward, 5′-GAAGGTGAAGGTCGGAGTC-3′; reverse, 5′-GAAGATGGTGATGGGATTTC-3′).

### Immunoblotting

Yeast cells transformed with pBT3-STE, pBT3-STE-CLDN7 were grown in selective media (SD-L). Total cell and tissue lysates were prepared using sodium dodecyl sulfate (SDS) buffer containing 62.5 mM Tris-HCl (pH 6.8), 10% glycerol and 2% SDS. Protein levels were quantified by bicinchoninic acid assay (BCA) assay kit (Pierce, IL, USA). 10 µg of total proteins were separated by 10–20% SDS-PAGE (Tris-glycine gels; Invitrogen) and transferred to polyvinylidene difluoride membranes (Millipore, MA, USA). After blocking with 5% nonfat dry milk, membranes were washed with Tris-buffered saline containing 0.05% Tween-20 (v/v) and incubated overnight with primary antibodies against CLDN7, APLP2, LexA-VP16, GAPDH and beta-actin. Next day, membranes were washed and incubated with horseradish peroxidase-conjugated secondary antibody (antimouse or antirabbit IgG, 1:10,000; Amersham Biosciences Corp., NJ, USA). Immunoblots were developed using the enhanced chemiluminescence kit (ECL; Amersham Biosciences Corp.). GAPDH was used as a normalizing control in cell lines. Due to variability in the GAPDH levels in tissue samples, B-actin was used as a normalizing control. Data is representative of results observed in more than three sets of different experiments performed in duplicates.

### Antibodies

Rabbit polyclonal CLDN7 (Cat#34-9100) was purchased from Zymed Laboratories Inc (CA, USA). Mouse monoclonal GAPDH (6C5) were obtained from Abcam (Cambridge, UK). Rabbit polyclonal APLP2 (Cat#128603) and mouse monoclonal beta-actin (AC15) antibodies were obtained from Abcam. Mouse monoclonal antibodies directed against LeX A were obtained from Dualsystems Biotech. Peroxidase-linked donkey antirabbit immunoglobulin and sheep antimouse IgG horseradish antibodies were obtained from Amersham Biosciences (GE Healthcare, NJ, USA). Alexa fluor antibodies were purchased from Molecular Probes (Thermo Fisher).

### *In silico* analysis

Proteins identified in yeast two-hybrid systems were further analyzed by Ingenuity pathway analysis tool to determine their interaction with CLDN7 (QIAGEN Inc., www.qiagenbioinformatics.com/products/ingenuity-pathway-analysis).

### Survival assay

OVCA420 cells were transfected with control siRNA or APLP2 siRNA. Twenty four hour post-transfection, 500–5000 cells were plated in a six-well dish in McCoy’s 5A growth medium (Invitrogen), supplemented with 10% fetal bovine serum and antibiotics (100 units/ml penicillin and 100 mg/ml streptomycin). Cells were incubated for a week at 37°C in CO_2_ incubator, stained with crystal violet and numbers of colonies were counted manually. All assays were performed in triplicates and data represents +/− SEM.

## Results

### Split ubiquitin membrane-based yeast two-hybrid system identified 30 protein interacting with CLDN7

**Figure 1.**
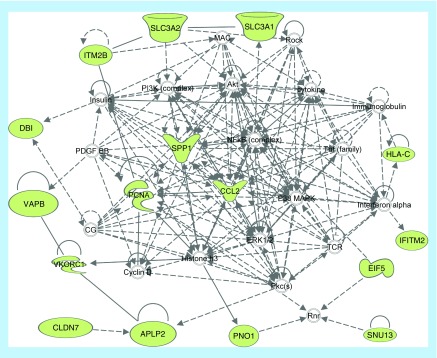
Network analysis of CLDN7 interacting protein. A set of 16 proteins identified in our yeast two-hybrid screen was analyzed by Ingenuity pathway analysis to determine direct or indirect protein–protein interactions. Protein interaction networks were developed based on the information stored in Ingenuity Pathway Knowledge Base.

Transformation of human kidney cDNA library in NMY51 cells expressing the bait vector (pBT3-STE-CLDN7-Cub-LexA-VP16), followed by growth on selective media identified 30 proteins interacting with CLDN7 (Table 1). Network analysis of CLDN7 interacting protein using Ingenuity pathway analysis, demonstrated both direct and indirect interactions of 16 proteins identified in our yeast two-hybrid screen (Figure 1). Ingenuity Pathway Analysis (IPA) also identified an indirect interaction of CLDN7 and APLP2.

**Table 1.** List of genes interacting with CLDN7 identified using yeast split ubiquitin system for membrane proteins.

Serial number	Entrez gene name	Gene symbol	Location
1.	Amyloid beta precursor-like protein 2	*APLP2*	Cytoplasm
2.	CD63 antigen isoform A	*CD63*	Plasma membrane
3.	C–C motif chemokine ligand 2	*CCL2*	Extracellular space
4.	Cornichon family AMPA receptor auxiliary protein 4	*CNIH4*	Plasma membrane
5.	Diazepam binding inhibitor, acyl-CoA binding protein	*DBI*	Cytoplasm
6.	Eukaryotic translation initiation factor 5	*EIF5*	Cytoplasm
7.	F-box protein 44	*FBXO44*	Cytoplasm
8.	Growth hormone inducible transmembrane protein	*GHITM*	Cytoplasm
9.	G protein subunit beta 2	*GNB2*	Plasma membrane
10.	HEAT repeat containing 5A	*HEATR5A*	Other
11.	Major histocompatibility complex, class I, C	*HLA-C*	Plasma membrane
12.	Major histocompatibility complex, class II, DP-α-1	*HLA-DPA1*	Plasma membrane
13.	Interferon-induced transmembrane protein 2	*IFITM2*	Cytoplasm
14.	Integral membrane protein 2B	*ITM2B*	Plasma membrane
15.	Proliferating cell nuclear antigen	*PCNA*	Nucleus
16.	Phosphatidylinositol glycan anchor biosynthesis class K	*PIGK*	Cytoplasm
17.	Partner of NOB1 homolog	*PNO1*	Nucleus
18.	Ribonuclease K	*RNASEK*	Other
19.	Signal peptidase complex subunit 1 homolog	*PCS1*	Other
20.	Ribosomal protein L3	*RPL3*	Nucleus
21.	Solute carrier family 39 member 1	*SLC39A1*	Plasma membrane
22.	Solute carrier family 3 member 1	*SLC3A1*	Plasma membrane
23.	Solute carrier family 3 member 2	*SLC3A2*	Plasma membrane
24.	SLU7 homolog, splicing factor	*SLU7*	Nucleus
25.	Small nuclear ribonucleoprotein 13	*NHP2L1*	Nucleus
26.	Secreted phosphoprotein 1	*SPP1*	Extracellular space
27.	Thioredoxin-related transmembrane protein 2	*TMX2*	Other
28.	Tubulin α-1c	*TUBA1C*	Cytoplasm
29.	Vesicle Associated Membrane Protein-associated protein B and C	*VAPB*	Plasma membrane
30.	Vitamin K epoxide reductase complex subunit 1	*VKORC1*	Cytoplasm

In a previous study [6], siRNA-mediated knockdown of CLDN7 resulted in upregulation of APLP2 mRNA. Since APLP2 was also identified in our yeast two-hybrid screen, we decided to focus on APLP2 for further study.

### APLP2 expression is increased in ovarian cancer

**Figure 2.**
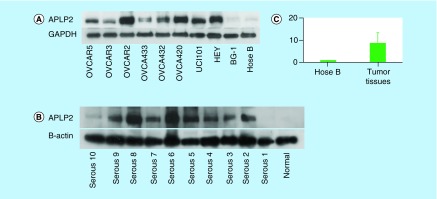
Expression levels of APLP2 protein in ovarian cancer cell lines and ovarian tumor tissue samples. Levels of APLP2 protein in ovarian cancer cell lines **(A)** and tumor tissue samples **(B)** and levels of APLP2 messenger RNA in tumor tissue samples **(C).** GAPDH and B-actin were used as a loading control for cell lines and tissues, respectively. Ovarian cancer cell lines and tumor tissue samples stained with APLP2 show strong staining compared with normal human ovarian surface cell line (HOSE B) and normal ovarian tissue (non-neoplastic tissue). All ovarian tumor tissues exhibited higher level of APLP2 messenger RNA compared with normal human ovarian surface cell line (HOSE B).

Expression of APLP2 protein was increased in all ovarian cancer cell lines, except BG-1, when compared with the normal human epithelial cell line, HOSE-B. The difference in expression levels of APLP2 among different cell lines might be due to the heterogeneous nature of ovarian cancer. Increased expression of APLP2 messenger RNA and protein was also found in ovarian tumor tissue samples (Figure 2A, B & C).

**Figure 3.**
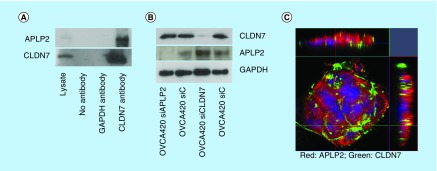
Expression levels of CLDN7, APLP2 proteins and their localization in OVCA420 cells. **(A)** Immunoprecipitation of CLDN7 and APLP2. OVCA420 cells were grown for 72 h and cell lysates were immunoprecipitated with anti-CLDN7, anti-APLP2 and anti-GAPDH antibodies. Proteins were visualized by immunoblotting with an anti-APLP2 and anti-CLDN7 antibodies. Total cell lysate serves as a positive control and no primary antibody in immunoprecipitation serves as a negative control. **(B)** Immunoblotting analysis of CLDN7 and APLP2 after siRNA-mediated knockdown of CLDN7 and APLP2. GAPDH was used as a loading control. **(C)** Immunofluorescence of OVCA420 cell line shows strong expression of CLDN7 mainly at cell junction and APLP2 expression at cell junctions as well as cytoplasm. CLDN7 and APLP2 also show colocalization at cell junctions. After fixing in methanol, OVCA420 cells were incubated with primary CLDN7 and APLP2 antibodies and visualized with secondary antibodies conjugated to Alexa fluor (Red color represents APLP2 and green color represents CLDN7). Nuclei were counterstained with DAPI.

To examine *in vivo* association of APLP2 and CLDN7, we performed immunoprecipitation using APLP2 or CLDN7 antibodies with lysates prepared from the OVCA420 cell line, which show endogenous expression of both CLDN7 and APLP2. APLP2 was precipitated with CLDN7 in IP with CLDN7 antibody, but not with control IgG antibody or a GAPDH antibody (Figure 3A). However, CLDN7 was not detected in IP with APLP2 antibody. A possible reason may be that CLDN7 association with APLP2 blocks the APLP2 epitope recognized by the antibody. SiRNA-directed downregulation of CLDN7 led to an increased expression of APLP2; however, knockdown of APLP2 did not affect CLDN7 expression (Figure 3B) suggesting a regulatory role of CLDN7 on APLP2 expression. Immunofluorescence analysis demonstrated partial colocalization of APLP2 and CLDN7 in the OVCA420 cell line, further supporting a possible direct interaction between these two proteins (Figure 3C).

### APLP2 downregulation decreased survival of ovarian cancer cells

**Figure 4.**
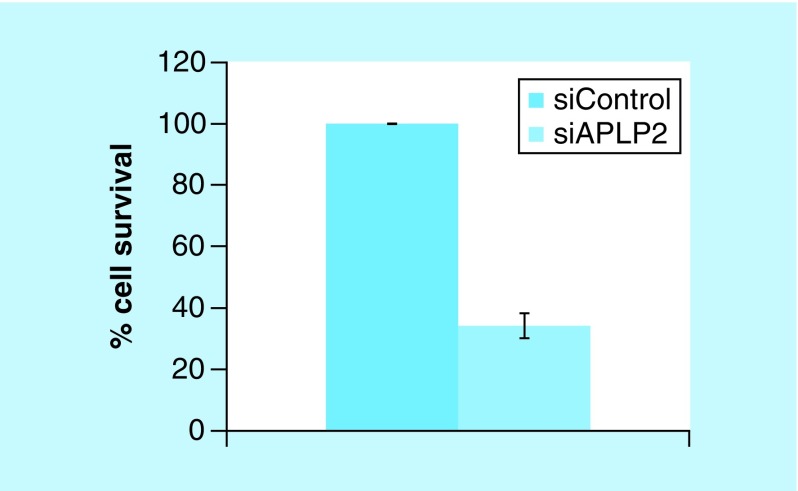
Cell survival analysis of OVCA420 cells after siRNA knockdown of APLP2. OVCA420 cells were treated with APLP2 siRNA and cell survival ability was determined by counting number of colonies formed after a 1 week incubation at 37°C in a humidified CO_2_ incubator. The data is representative of three independent experiments.

In order to investigate functional role of APLP2 in ovarian cancer, we studied effect of APLP2 knockdown on cell survival ability of OVCA420 cells. OVCA420 cells transfected with siAPLP2 showed a significant decrease in survival compared with control siRNA-treated cells, indicating a significant role of APLP2 in ovarian cancer cell survival (Figure 4).

## Discussion

Claudins are tight junction proteins on the apical side of the cells, which control cell polarity and barrier function. The tightness of the junction is determined by the combinations of different claudin proteins involved in formation of that specific junction. Deregulation of tight junctions have been linked to several disease conditions, such as Crohn’s disease [13], lung disease [14], celiac disease [15], viral infections [16], capillary amyloid angiopathy [17] and cancer [3,18]. Deregulation of claudin proteins have been reported in many human cancers, including ovarian cancer [3,6,19,20]. In particular, CLDN7 has been shown to be deregulated in ovarian cancer [4,6].

Overexpression of CLDN7 has been correlated with poor progression-free survival in ovarian cancer [21,22]. Ovarian cancer showing higher expression of CLDN7 demonstrated lower sensitivity to platinum-based drugs, with increased sensitivity after knockdown of CLDN7, suggesting a direct role of CLDN7 in inducing drug resistance in ovarian cancer cells [21]. In our previous study, we highlighted that upregulation of CLDN7 was associated with increased invasion of cancer cells, suggesting a physiological role of CLDN7 in ovarian carcinoma [6]. In addition, modulation of CLDN7 using siRNA altered the expression of several genes at the mRNA and protein level.

To find possible interacting partners of CLDN7, we applied the split ubiquitin-based yeast two-hybrid system for membrane proteins [23]. Our yeast two-hybrid system identified 30 interacting partners of CLDN7. Among different interacting partners, APLP2 was particularly interesting as our previous report demonstrated that the changes in expression of APLP2 mRNA in CLDN7 siRNA transfected ovarian cancer cell line. Here, we observe an increase in the APLP2 protein level after knockdown of CLDN7 in OVCA420 cells. The direct interaction of APLP2 with CLDN7 was further confirmed by immunoprecipitation where APLP2 was found directly associated with CLDN7.

This is the first report on the direct association of CLDN7 with APLP2. There are few reports on direct involvement of amyloid precursor proteins in cancer. Increased expression of amyloid precursor protein has been reported in pancreatic cancer [24–26] gastrointestinal tumors [27]. Along with amyloid precursor protein, APLP2 was frequently expressed in gastrointestinal tumors [27]. Increased expression of APLP2 in several cancers and its role in migration [28], wound healing [29] and cellular proliferation [30] is indicative of its important functions in cancer. Increased expression of APLP2 in ovarian cancer and decreased survival of ovarian cancer cells after siRNA-mediated knockdown of APLP2 suggest potential role of APLP2 in ovarian cancer pathogenesis.

## Conclusion

In summary, this is the first study reporting interaction of CLDN7 with APLP2 and effect of APLP2 on cell survival in the ovarian cancer. This study provides qualitative information only. Further studies are warranted to understand the interplay between CLDN7 and APLP2 and their involvement in cancer development and progression.

## Future perspective

The yeast two-hybrid system for membrane protein is a powerful tool to identify proteins interacting with each other. Our data identified direct interaction of CLDN7 and APLP2 in ovarian cancer and effect of APLP2 on ovarian cancer cell survival. Future studies may include work on identifying the mechanism involved in APLP2-mediated ovarian cancer cell survival as well as finding other interacting partners of CLDN7 and their role in ovarian cancer.

**Summary points**

**Aim**

- Downregulation of CLDN7 resulted in increased levels of several messenger RNAs including APLP2 messenger RNA in ovarian cancer cell lines.
- This study was performed to identify proteins interacting with CLDN7 which might be responsible for increased expression of messenger RNAs reported in our earlier study.

**Materials & methods**

- The yeast two-hybrid system was used to find interacting partner of CLDN7 using ovarian cancer cell line OVCA420 as a model.
- Selected candidates identified in yeast two-hybrid screen were analyzed using Ingenuity pathway analysis to find their interaction with CLDN7.
- SiRNA-mediated approach was used to study effect of APLP2 on cancer cell survival.

**Results**

- Silencing of CLDN7 resulted in increased expression of APLP2 in ovarian cancer cell lines.
- Ovarian cancer tissue samples showed increased levels of APLP2 proteins compared with normal ovarian tissues (non-neoplastic tissue).
- Silencing of APLP2 decreased survival of ovarian cancer cell lines.

**Conclusion**

- APLP2 interacts with CLDN7 in ovarian cancer cells and affect ovarian cancer cell survival.
